# Perceived Stress During the First Wave of COVID-19 Outbreak: Results From Nationwide Cross-Sectional Study in Estonia

**DOI:** 10.3389/fpubh.2021.564706

**Published:** 2021-06-18

**Authors:** Rainer Reile, Lembe Kullamaa, Reeli Hallik, Kaire Innos, Maarja Kukk, Kaia Laidra, Eha Nurk, Merili Tamson, Sigrid Vorobjov

**Affiliations:** ^1^Department of Epidemiology and Biostatistics, National Institute for Health Development, Tallinn, Estonia; ^2^Institute for Family Medicine and Public Health, University of Tartu, Tartu, Estonia; ^3^Department of Nutrition Research, National Institute for Health Development, Tallinn, Estonia; ^4^Department of Drug and Infectious Diseases Epidemiology, National Institute for Health Development, Tallinn, Estonia

**Keywords:** COVID-19, SARS-CoV-2, pandemic, mental health, stress, Estonia

## Abstract

**Objective:** To study the population-level mental health responses during the first wave of coronavirus disease 2019 (COVID-19) outbreak in Estonia and analyze its socio-demographic, behavioral, and health-related variations among general population.

**Methods:** This study used nationally representative data on 4,606 individuals, aged 18–79 years from a rapid-response cross-sectional survey conducted in April 2020. Point prevalence and mutually adjusted prevalence rate ratios for perceived stress from log-binomial regression analysis were presented for socio-demographic, behavioral, and health-related variables.

**Results:** This study found that 52.2% of population aged 18–79 reported elevated stress levels in relation to COVID-19 outbreak. Higher levels of perceived stress were found in women, in younger age groups, in Estonians, and in those with higher self-perceived infection risk, presence of respiratory symptoms, and less than optimal health, according to self-reports.

**Conclusion:** Although, the potential long-term health effects of the current crisis are yet unknown, the alarmingly high stress levels among people indicate that the COVID-19 pandemic might have had a widespread effect on people's mental health.

## Introduction

The ongoing outbreak of novel coronavirus SARS-CoV-2 started in December 2019 with the first documented cases of pneumonia of unknown origin registered in Hubei province, China (1). Despite the efforts to contain the virus locally, it spread rapidly across the world, and on March 11, 2020, the coronavirus disease 2019 (COVID-19) outbreak was labeled a pandemic by the World Health Organization. As of mid-March 2021, 120 million cases have been confirmed globally, and the estimated death toll exceeds 2.6 million (2).

While the acute health burden of COVID-19 is undoubtedly heavy, the wider health outcomes of the pandemic might be even more widespread. Evidence from earlier epidemics (e.g., SARS in 2003 or the H1N1 pandemic in 2009) suggests widespread mental health impacts (3–5). People who have been directly infected or those in direct contact with someone with the infection may experience increased stress and anxiety, depression, and also post-traumatic stress disorder (6, 7), but the perceived infection risk could also lead to higher anxiety among non-infected individuals (8). Given the short timeframe, only a limited number of studies have so far analyzed the mental health consequences of the current pandemic. However, the evidence from very recent systematic reviews (9, 10) confirms higher levels of mental health problems during the COVID-19 pandemic. Regardless of the study population, a higher psychological impact of COVID-19 is reported for females, for those having lower socioeconomic status, and for those at higher risk of contracting COVID-19 due to poor health or contact with COVID-19 patients (10).

**Figure 1:**
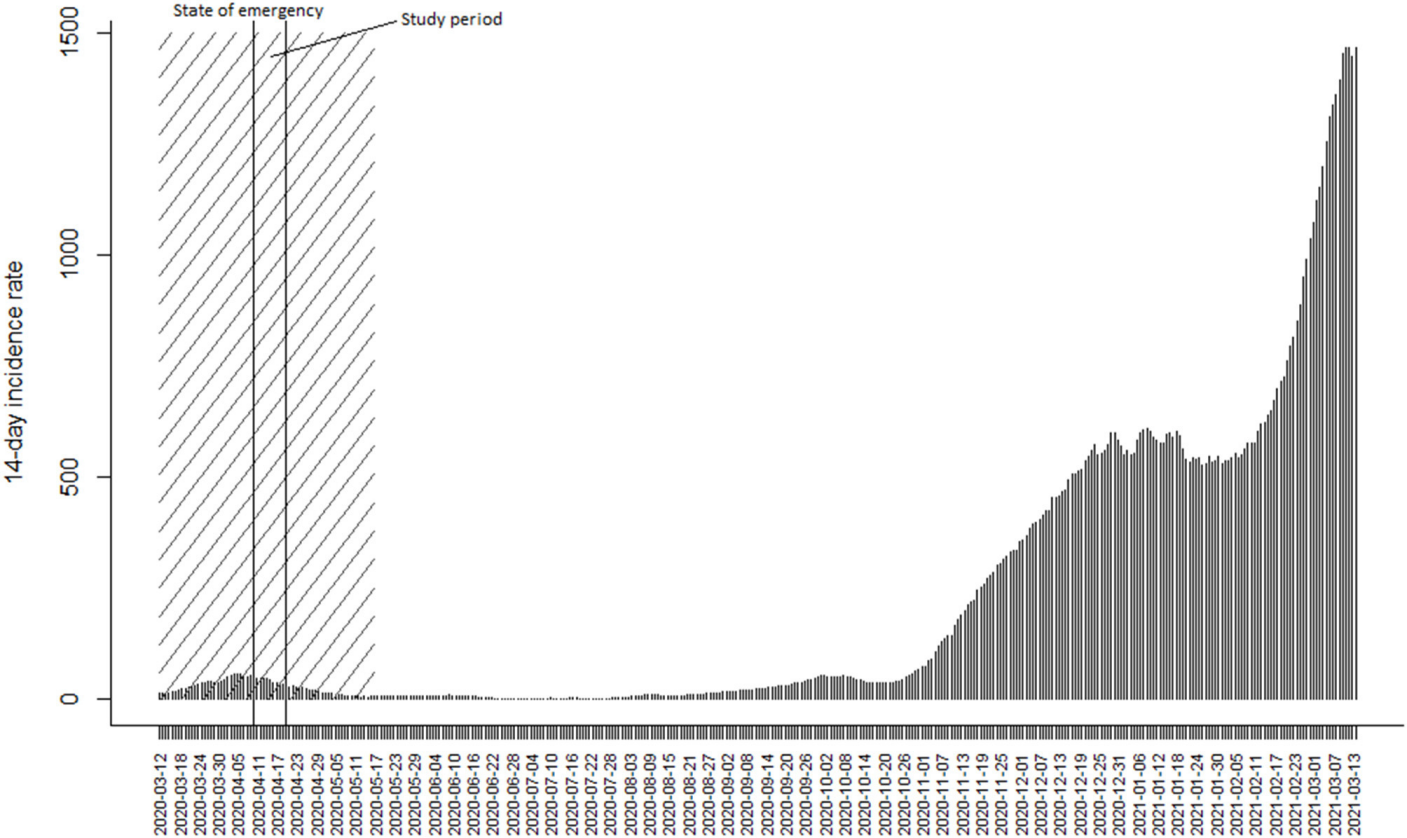
The 14-day incidence rate for COVID-19 cases per 100,000 over a 1-year period in Estonia.

This paper contributes to the field by covering the immediate mental health responses to the COVID-19 outbreak using a nationally representative dataset. More specifically, we will focus on the prevalence of perceived stress during the first wave of the pandemic in Estonia, where the first COVID-19 case was confirmed on February 25, 2020 (11). With 58 cases diagnosed, a state of emergency was declared by the government on March 12, 2020, to enforce appropriate measures to control the spread of infection. During the first wave, the 14-day incidence peaked on April 6 at 56.6 cases per 100,000 and after gradual decline in newly diagnosed cases, the state of emergency was ended on 17th May 2020 with 14-day incidence being 6.0 per 100,000 (Figure 1). By end of May 2020, ~5% of the population had been tested and fewer than 2,000 COVID-19 cases had been confirmed in total (11). However, Figure 1 also illustrates the situation 10 months later when the ongoing second wave resulted in almost 2,000 new cases daily and the 14-day incidence rate was close to 1,500 per 100,000 as of mid-March 2021.

Although, the current epidemiological situation is in a stark contrast with the first wave of the COVID-19 pandemic in Estonia, several aspects suggest that first wave may have had considerable impact on mental health despite the relatively modest incidence. First, the evolving pandemic saw extensive attention in all media platforms that, coupled with the overall novelty of the situation, may have resulted in increased perception of fear and anxiety (12). Second, the emergency situation itself and the measures taken to contain the spread of infection (13) were unprecedented and affected the daily lives of all inhabitants. In view of this, the aim of the study was to analyze the prevalence of perceived stress and its patterning across sociodemographic, behavioral, and health-related covariates during the first wave of the COVID-19 outbreak in Estonia.

## Methods and Materials

### Data

This study uses data from a nationwide rapid-assessment survey conducted during the peak of the first wave of the pandemic in Estonia when the hospitalization rates were at their highest and most restrictions had been enforced. A nationally representative stratified random sample of 12,000 individuals aged 18–79 years with a valid email address was obtained from Population Registry for the survey. The survey questionnaire was based on locally adapted version of WHO tool for behavioral insights on COVID-19 (14) with additional items on the presence of symptoms of upper respiratory infections, previous COVID-19 testing, self-rated health (SRH), and perceived stress. In total 4,606 responses were submitted during the 10-day study period between April 10 to April 20, 2020. After accounting for 558 cases who were unable to respond due to invalid email addresses, the adjusted response rate was 40.3%. Population weights based on age and gender distribution of the Estonian population were used to adjust for oversampling of youngest and oldest age groups and to compensate for the non-response. The study protocol was approved (no. 271, from April 8, 2020) by the Research Ethics Committee of the National Institute for Health Development.

### Variables

The dependent variable was self-reported perceived stress that was assessed with the question “Are you currently experiencing stress or anxiety?” The response options were dichotomized as (i) excessive stress (“yes, much more than previously” and “yes, somewhat more than previously”), and (ii) not stressed (“yes, but not more than previously” and “no, not at all”). The same instrument has been used in several national health surveys previously and thus provides a valid comparison.

Respondents' demographic backgrounds were described by variables of gender, age, education, ethnicity, and place of residence. Age effects were analyzed in age groups of (i) 18–34, (ii) 35–49, (iii) 50–64, and (iv) 65–79. Educational level was measured by the highest level of education obtained and dichotomized into categories of (i) up to secondary or vocational and (ii) tertiary education. Self-reported ethnicity was grouped as (i) Estonians and (ii) non-Estonians, referring to other, mostly Russian-speaking ethnic groups. Respondents' places of residence were dichotomized as (i) rural and (ii) urban areas.

Additional behavioral and health-related items included self-perceived infection risk, conforming to isolation measures, presence of respiratory symptoms, and SRH. Subjective infection risk was assessed with a binary (yes/no) question: “Do you think you are likely to become infected with the novel coronavirus?” Responses to questions “Are you currently in isolation (do you avoid social contact)?” and “Have you experienced symptoms typical of upper respiratory infections since the beginning of March 2020?” were used to assess conformity to isolation measures and presence of respiratory symptoms, respectively. SRH was covered with a single-item question, “How would you assess your present state of health?” with response options dichotomized into the categories (i) average or poor and (ii) good health.

### Analysis

The prevalence of stress was calculated as the proportion of cases reporting excess stress divided by the total number of cases by sociodemographic variables, with 95% confidence intervals (95% CI). To study the variations between stress and independent variables, log-binomial regression with robust variance estimates was used for the analysis. This approach avoids the overestimation of the association that is often found for logistic regression when the outcome is frequent (15, 16). The results are presented as exponentiated coefficients from a mutually adjusted model that are interpreted as prevalence ratios (PR) with 95% confidence intervals. All statistical analyses were conducted using SPSS Statistics for Windows, version 25.0 (IBM Corp. 2017). A *p*-value <0.05 was regarded as statistically significant.

## Results

In total, 52.2% of the respondents felt excessive stress e.g., were currently more anxious or stressed than previously (Table 1). The prevalence of stress was higher among women, in younger age groups, Estonians and among respondents with higher self-perceived infection risk, presence of respiratory symptoms, and less-than-good SRH.

**Table 1:** Prevalence rates and adjusted prevalence ratios with 95% confidence intervals for excessive stress.

		**Total sample (*****n*** **=** **4,606)**		**Prevalence**	**Prevalence ratio*^a^***
		***N***	**%**	**% (95% CI)**	**PR (95% CI)*^b^***
Gender	Women	2,396	52.2	58.2 (56.3–60.2)	**1.09 (1.07–1.11)**
	Men	2,190	47.8	45.5 (43.4–47.6)	1
Age	18–34	1,271	27.8	55.3 (52.6–58.0)	**1.12 (1.08–1.15)**
	35–49	1,267	27.6	54.9 (52.2–57.7)	**1.10 (1.06–1.13)**
	50–64	1,182	25.8	50.9 (48.1–53.8)	**1.06 (1.03–1.09)**
	65–79	861	18.8	45.1 (41.8–48.4)	1
Education	≤ Secondary/vocational	2,695	58.8	51.1 (49.2–53.0)	0.99 (0.97–1.01)
	Tertiary	1,889	41.2	53.6 (51.4–55.9)	1
Ethnicity	Estonian	3,723	81.2	53.4 (51.8–55.0)	**1.05 (1.02–1.07)**
	Other	861	18.8	46.9 (43.6–50.3)	1
Place of residence	Rural	1,194	26.0	49.7 (46.8–52.5)	0.99 (0.97–1.01)
	Urban	3,390	74.0	53.0 (51.4–54.7)	1
Perceived infection risk	Yes	2,354	53.0	57.6 (55.6–59.6)	**1.07 (1.05–1.09)**
	No	2,088	47.0	45.7 (43.6–47.8)	1
Being in isolation	Yes	2,376	52.5	52.9 (50.9–54.9)	1.00 (0.98–1.02)
	No	2,148	47.5	51.5 (49.4–53.6)	1
Respiratory symptoms	Yes	663	14.5	61.1 (57.5–64.9)	**1.02 (1.00–1.05)**
	No	3,909	85.5	50.7 (49.1–52.3)	1
Self–rated health	Average or poor	1,029	22.5	63.1 (60.1–66.0)	**1.12 (1.09–1.14)**
	Good	3,548	77.5	49.0 (47.3–50.6)	1

a *Adjusted for all covariates listed*.

b *Statistically significant (p < 0.05) associations are given in boldface*.

In the mutually adjusted regression model, excess stress was significantly higher among women compared to men (PR 1.09; 95% CI 1.07–1.11), in younger age groups compared with 65- to 79-year-olds, and among Estonians compared to non-Estonians (PR 1.05; 95% CI 1.03–1.09). As with crude prevalence, the respondents' education or place of residence did not differentiate stress levels. However, those with higher self-perceived infection risk, presence of respiratory symptoms, and less-than-good SRH had higher stress levels.

## Discussion

### Main Findings

This study found that over half of the population experienced excess stress with higher stress prevalence found among women compared with men, in age groups below 65 years compared with 65- to 79-year-olds, and among Estonians compared with non-Estonians. In addition to these sociodemographic factors, higher stress was reported by those with higher self-perceived infection risk, presence of respiratory symptoms, and less-than-good SRH. With slight variations, these findings are generally in accordance with previous evidence (10) on the sociodemographic patterning of mental health outcomes during COVID-19 pandemic.

The alarmingly high stress levels found in current data are in stark contrast with earlier data from 2018 (17), where similar stress indicators were reported by 18.7% of men and 20.7% of women (19.9% in total). Also, the current stress prevalence is more than 2-fold compared with data from the previous economic recession in 2010 (17). As economic recessions lead to rises in unemployment levels and reductions in staff and wages are correlated with an increase in mood disorders, anxiety, depression, and suicide (18), it is very likely that both the direct epidemiological emergency and its wider social and economic consequences have already translated into an increase in mental health problems as suggested by a few recently published studies (19, 20).

The demographic patterning of stress indicates that some sociodemographic groups were more affected than others. Similarly to a recent study (21), a distinct age gradient was found, with the highest stress being reported in the youngest age group, despite the evidence that COVID-19 presents a higher health risk for those aged 65 and older (22). This also contradicts earlier evidence on the mental health effects of the economic crisis from Estonia in the late 2000's (23), when perceived depression had increased most in ages 35 and up. However, the causes and the consequences of both crises are very different. As the older generations have experienced stressful life events (e.g., the post war period, Soviet repression, the struggle for independence, and extreme economic difficulties during the 1990's) (24), it is plausible that the current state of emergency with its unprecedented social distancing measures (25) could affect younger age groups the most. Such a negative psychological impact has also recently been demonstrated among undergraduates in the context of the COVID-19 pandemic (26). The gender differences in perceived stress found in our data are supported by earlier evidence that women are generally more vulnerable to stress- and fear-based disorders (27). However, a range of other potential explanations could be relevant in the current context as well. Women more often work in healthcare, the service sector, and other high-risk occupations in the context of COVID-19. Several studies have shown higher levels of mental health problems in health care workers and in customer service during the current pandemic (28, 29). Moreover, the school closures and social distancing measures could put additional strain on women due to increase in tasks related to childcare, housework and caring for the sick (30). The higher stress prevalence seen in Estonians compared with other, mostly Russian-speaking ethnic groups is consistent with observed long-term trends in perceived stress and depression (17). This could also suggest that the health communication during the current pandemic has not preferred the majority ethnic group over minorities.

In addition to demographic indicators, several behavioral and health-related variables differentiated stress levels in our data. As the COVID-19 pandemic coincides with the period of seasonal influenza and other upper respiratory infections, self-perceived infection risk and presence of symptoms were both expectedly associated with higher stress. As the most common symptoms of COVID-19 are fever, cough, fatigue, and shortness of breath (31), having similar symptoms could lead to higher anxiety and stress levels. Similarly, those with average or poor SRH had 12% higher prevalence of excessive stress compared with respondents with good SRH. SRH is a valid estimate for overall health status that has strong predictive power for future health outcomes (32). SRH follows the age gradient, and poor health is often associated with chronic conditions and comorbidities (33) that constitute higher health risk also in the context of COVID-19 (22).

The main strength of the study is the timing of the nationally representative survey that was purposely designed to assess the knowledge and public perception during the first wave of the COVID-19 pandemic. However, some potential limitations of the study need to be addressed. First, the survey relied on a self-assessed, single-item measure of stress and anxiety. Although, it is not sufficient for a clinical diagnosis, it reflects the subjective presence of anxiety and stress-related complaints and allows comparisons with previous health surveys in Estonia. Second, due to the state of emergency, the survey was conducted using a web questionnaire only. Therefore, the representative sample of adults had to include only individuals with valid email addresses in the population registry database. Although, earlier studies (17) have shown that ~90% of individuals have valid email addresses in the population registry database, a potential selection bias cannot be fully excluded in our data. Third, the cross-sectional data do not allow us to establish causality *per se*. Despite this, the time anchoring of the dependent variable provides relevant estimates with respect to the time frame of the current the study. Moreover, the study protocol has been amended to allow additional data collection from the same sample, thus providing an opportunity for a longitudinal design in further studies. Fourth, due to the short data collection period, the response rate was modest (40.3%). Finally, despite the inclusion of different demographic, health-related, and behavioral variables, it is unlikely that the set of variables accounts for the total variance of the dependent variable. Thus, potential residual bias should be considered when interpreting the results. However, the large sample size and the use of population weights to reduce the potential non-response bias assure the representativeness of the data.

### Conclusions

With over half of the 18- to 79-year-olds experiencing excess stress or anxiety during the first wave of COVID-19 pandemic in Estonia, the potential mental health impacts of the pandemic cannot be ignored. Although, direct causality cannot be established, the underlying uncertainty regarding the social, political, and economic aftermath of the pandemic is potentially said to have widespread negative effects on a population's mental health. Moreover, the long-term effects of current crisis are yet unknown. Further longitudinal studies are therefore needed to assess whether the high stress levels translate into acute or chronic (mental) health problems that could place additional strain on the health sector and affect public health outcomes in general. Close monitoring of the mental health outcomes is therefore warranted to ensure timely access to mental healthcare.

## Data Availability Statement

The raw data supporting the conclusions of this article will be made available by the authors, without undue reservation.

## Ethics Statement

The studies involving human participants were reviewed and approved by Research Ethics Committee of the National Institute for Health Development. Written informed consent for participation was not required for this study in accordance with the national legislation and the institutional requirements.

## Author Contributions

All authors listed have made a substantial, direct and intellectual contribution to the work, and approved it for publication.

## Conflict of Interest

The authors declare that the research was conducted in the absence of any commercial or financial relationships that could be construed as a potential conflict of interest.
